# Seq-ing answers: uncovering the unexpected in global gene regulation

**DOI:** 10.1007/s00294-018-0839-3

**Published:** 2018-04-19

**Authors:** George Maxwell Otto, Gloria Ann Brar

**Affiliations:** Department of Molecular and Cell Biology, University of California-Berkeley, Berkeley, CA 94720 USA

**Keywords:** Gene regulation, Gene expression, mRNA-seq, Non-canonical gene expression, LUTI, Transcription, Translation, Ribosome profiling, Mass spectrometry, Transcript isoforms, uORFs

## Abstract

The development of techniques for measuring gene expression globally has greatly expanded our understanding of gene regulatory mechanisms in depth and scale. We can now quantify every intermediate and transition in the canonical pathway of gene expression—from DNA to mRNA to protein—genome-wide. Employing such measurements in parallel can produce rich datasets, but extracting the most information requires careful experimental design and analysis. Here, we argue for the value of genome-wide studies that measure multiple outputs of gene expression over many timepoints during the course of a natural developmental process. We discuss our findings from a highly parallel gene expression dataset of meiotic differentiation, and those of others, to illustrate how leveraging these features can provide new and surprising insight into fundamental mechanisms of gene regulation.

## Introduction

The canonical model for gene expression, whereby information in genomic DNA sequences is decoded to produce protein through an mRNA intermediate, was defined based on painstaking studies by many labs using single-gene manipulations and biochemical approaches. Subsequent studies over decades identified additional complexity—including specificity factors and mRNA processing steps—that have added to our understanding of how gene expression is more-or-less universally regulated in eukaryotes. The development of techniques for global gene expression measurement within the last two decades was invaluable in enabling broad interrogation of these pre-existing “rules” for how gene regulation works. Now, with the widespread use of such methods, we can precisely measure every step in gene expression genome-wide. Employing these measurements has accelerated discovery in gene expression, in some cases confirming existing models, and sometimes revealing surprisingly common types of “non-canonical” regulation.

Genome-scale experiments provide immense amounts of data that can be analyzed to identify trends at individual levels of gene regulation and to highlight exceptional cases. Here, we discuss our recent study (Cheng et al. 2018), which used parallel global gene expression measurements to identify poor correlation between mRNA and protein levels over time for hundreds of genes controlled by an unconventional mode of gene regulation. For many genes regulated by this mechanism, an apparent increase in transcription corresponded with a switch to a poorly translated transcript, ultimately leading to a decrease in protein produced from the encoded locus. We will highlight important features of our experimental approach that enabled this surprising finding and discuss other studies that have been similarly successful in leveraging complex datasets to reveal specific mechanistic insight into a range of cellular gene regulatory strategies.

## Our experiment: an overview

**Fig. 1:**
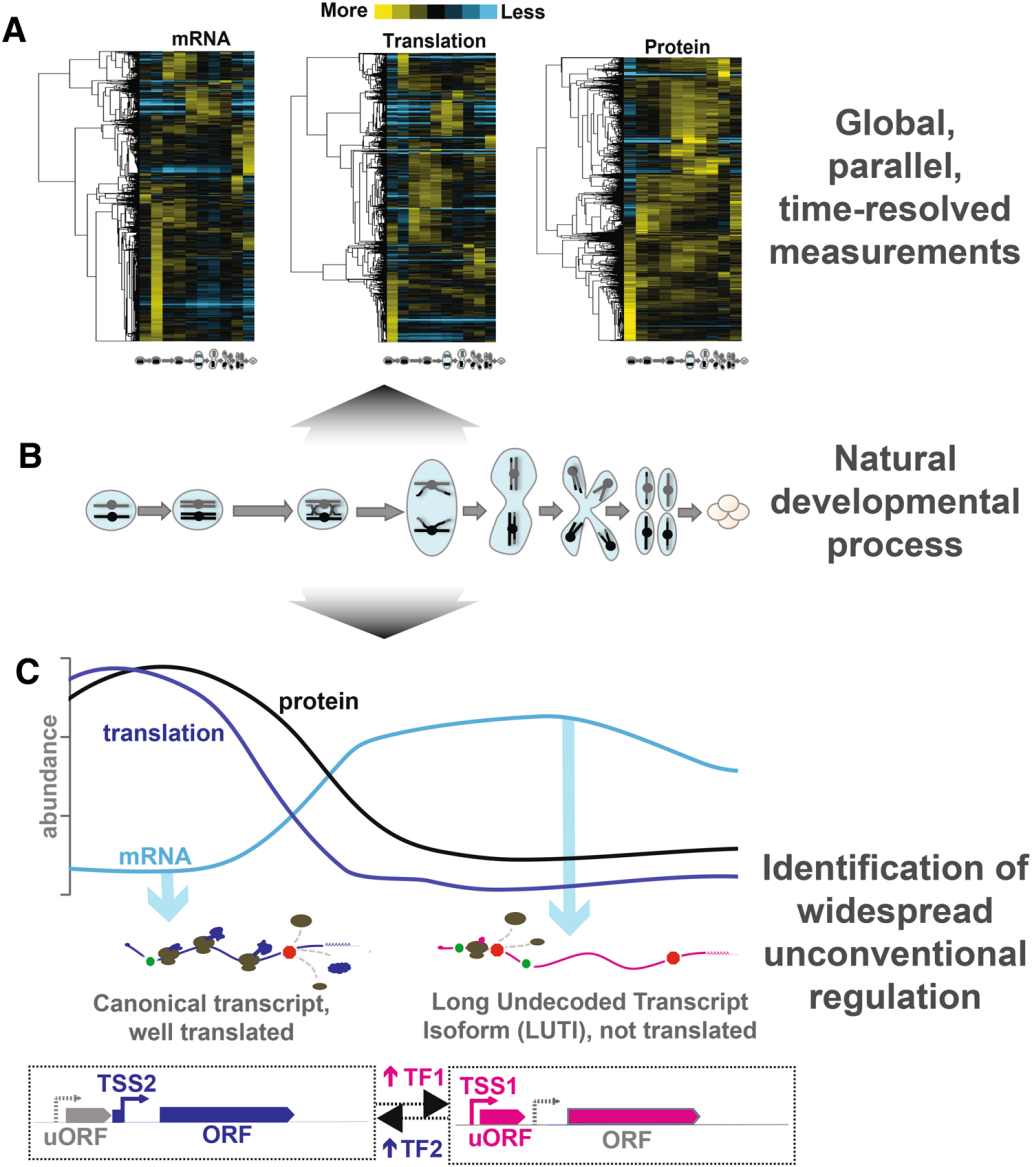
Integrated analysis of parallel, genome-wide measurements through meiotic development reveals pervasive non-canonical regulation of gene expression. **a** We performed RNA sequencing (mRNA), ribosome profiling (translation) and quantitative mass spectrometry (protein) on matched lysate from a timecourse spanning meiosis in budding yeast. Hierarchical clustering is displayed with rows representing individual genes (*n* = 4464) that were quantified across all measurements and timepoints. Columns represent timepoints through meiotic differentiation depicted in **b. b** Meiosis is a conserved cellular differentiation program comprised of a coordinated series of unidirectional transitions in cell state, ultimately producing haploid gamete cells from a diploid precursor. **c** Above, measurements of mRNA, translation and protein abundance are depicted for a model locus regulated by transcript isoform toggling. Below, a diagram for this model gene locus is shown. Transcription from an ORF-proximal transcription start site (TSS2) mediated by a developmentally regulated transcription factor (TF2) leads to the production of a canonical, well-translated transcript and the ORF-endcoded protein is abundant. A decrease in TF2 and an increase in another transcription factor (TF1) results in a switch to transcription from an ORF-distal transcription start site (TSS1), producing a long undecoded transcript isoform (LUTI) mRNA that is poorly translated. ORF-encoded protein is reduced, despite increased transcription from this locus. As a consequence, ORF-encoding transcript abundance and ORF-encoded protein abundance show poor correlation over time. Using integrated parallel measurements outlined in **a**, we identified widespread use of this unconventional regulatory mechanism for hundreds of genes

Our study (Fig. 1) sought to define gene expression patterns comprehensively through budding yeast meiosis, a natural and conserved developmental process. Towards this end, we performed RNA-seq, ribosome profiling, and quantitative mass spectrometry on matched samples from ten conditions: eight timepoints spanning meiotic differentiation, one exponentially growing mitotic sample, and one media-matched non-meiotic sample. We restricted our analyses to cases that we were able to quantify at every timepoint, and by every method, which represented over 70% of annotated genes. Based on depth and time resolution, we believe that this is the most comprehensive gene expression atlas for a developmental program to date (Cheng et al. 2018).

Analysis of our dataset confirmed known regulation and revealed evidence of much more regulation at the level of protein abundance than was previously appreciated. Our most exciting finding, however, was the existence of a large class of genes for which mRNA levels were poorly predictive of protein levels. While this class is difficult to explain by traditional gene regulatory models, it included one gene that provided a hint to the regulation of other members of the group. This gene, *NDC80*, encodes a kinetochore component that is crucial for the success of meiosis (Kim et al. 2013; Meyer et al. 2015; Miller et al. 2012). *NDC80* was recently found to be regulated by an unconventional strategy involving two transcript isoforms differing only in their transcription start sites—and, therefore, the length of their 5′ leaders (Chen et al. 2017; Chia et al. 2017). The longer transcript isoform contains several upstream open reading frames (uORFs) that are efficiently translated, resulting in reduced translation of the canonical ORF. The extended transcript isoform was named “LUTI” for long undecoded transcript isoform, and its transcription also resulted in reduced transcription of the canonical isoform at this locus (Chen et al. 2017; Chia et al. 2017). We queried our dataset for genes with features common to the LUTI-based regulation determined for *NDC80* and found 380 genes to be regulated by this mechanism, representing nearly 8% of all yeast genes for which we collected measurements (Cheng et al. 2018).

We further found that a single meiotic transcription factor could coordinately drive transcription of two distinct classes of targets—canonical transcripts at some loci and LUTIs at others—resulting in protein level increases or decreases, respectively (Cheng et al. 2018). This has interesting consequences for how we think about gene regulatory logic. A mechanism for coordinated protein up- and down-regulation may be particularly important in developmental contexts, in which protein function is often stage-specific and progression to the next stage may require not just new protein synthesis, but also attenuated production of proteins that are no longer required. Using a single transcription factor to both up- and down-regulate protein production from distinct sets of target genes seems an efficient way of achieving rapid and efficient transitions in cell state.

Key features of our experimental design enabled our findings. First, we required genome-scale measurements to identify classes of genes that were similarly regulated by this mechanism. Furthermore, our analysis was built on the ability to confidently measure correlation (and anti-correlation) between mRNA and protein, which in turn relied on genome-wide measurements taken in parallel and across many timepoints. Finally, our choice of experimental system, a natural process of cellular differentiation, allowed us to identify a mechanism that is ideal for rapid and coordinated temporal changes. Below we discuss in greater detail how we and others have leveraged these experimental features to gain important and perhaps surprising insight into gene regulatory processes genome wide.

## Genomics provides a wealth of information with emergent properties

The emergence of techniques for measuring global gene expression—beginning with microarrays in the 1990s—was revolutionary for the study of gene regulation (Brown and Botstein 1999). An unprecedented wealth of data allowed researchers to define near-complete transcriptional profiles for cells in any condition of interest [examples in Cho et al. (1998), Chu et al. (1998), DeRisi et al. (1997), Spellman et al. (1998)]. Researchers quickly realized that these datasets also possessed unforeseen emergent properties that enabled analyses beyond simply quantifying gene expression globally (Eisen et al. 1998; Zaslaver et al. 2004). For example, genes with shared function often cluster together based on expression patterns, enabling successful prediction of function for unstudied genes [reviewed in Brown and Botstein (1999)]. RNA sequencing later provided these same advantages while also allowing more detailed definitions of qualitative features, such as transcript boundaries and unbiased identification of transcription outside of predicted genes [reviewed in Berretta and Morillon (2009), Ozsolak and Milos (2011), Wang et al. (2009)]. Our study depended on an integrated analysis of both qualitative and quantitative aspects of genomic datasets. Without information about transcript boundaries together with abundance, for example, we could not have determined that the hundreds of new cases of unconventional protein regulation were based on transcript isoform toggling (Cheng et al. 2018).

Genome-wide measurements are powerful in that they inherently replicate every single-gene study ever performed in the condition of interest. By testing new datasets against the previous findings, researchers gain a straightforward quality control measure. If the data behave as predicted for cases in which regulation is known, it builds confidence in the dataset’s ability to identify novel phenomena. Studying thousands of genes in parallel allows researchers to identify trends and outliers, both of which are important for a full understanding of the biological process in question. Moreover, rather than requiring researchers to guess a suitable control gene for comparison to their query, genomic datasets have thousands of built-in controls. Our study, for example, relied on detection of many cases of canonical regulation (which showed high mRNA-to-protein correlations) to provide the contrast needed to reveal a large class of non-canonical cases (Cheng et al. 2018).

## Parallel measurements can capture the interplay between gene regulatory levels

It would be easy to dismiss disagreement between mRNA and protein levels as noise or measurement artifacts. We were nevertheless confident that there was biological meaning to the many cases of poor mRNA-to-protein correlation in our dataset, in part because our experimental design (Cheng et al. 2018) allowed direct comparison of mRNA and protein measurements from the same lysate, thus eliminating experiment-to-experiment variability. This type of preparation was also key to the first ribosome profiling studies, which enabled straightforward comparison of mRNA and translation levels (Brar et al. 2012; Ingolia et al. 2009, 2011). These experiments identified wider ranges in translation efficiencies (ribosome footprints/mRNA; TEs), than previously thought, suggesting unexpected translational specificity. Initial attempts to determine the relationship between mRNA and protein abundance relied on datasets from different labs and reported low apparent correlation between the two measurements. Selbach and colleagues (Schwanhäusser et al. 2011) were among the first to globally measure mRNA and protein abundance from the same sample, and found a much stronger mRNA-to-protein agreement (*R*^2^ = 0.41) than had been observed previously. However, single-sample comparisons—even among parallel samples—are largely driven by very highly and very lowly expressed genes. It is perhaps not surprising that the most highly abundant mRNAs also correspond to the most highly abundant proteins and does not necessarily preclude important post-transcriptional regulation. To illuminate additional regulation requires comparison, not only between different levels of gene regulation, but over time during conditions of cellular change.

## Regulatory dynamics captured by time-resolved series measurements

Our identification of protein-level regulation by widespread transcript toggling through meiotic differentiation depended on our ability to observe a poor or negative correlation between mRNA and protein over time (Cheng et al. 2018). We compared measurements from ten samples, giving us a high degree of confidence in our correlation values. It is impossible to make such determinations with a sample from a single condition. Comparing expression trends over time also overcomes bias in different measurement methods that may result in misinterpretation of data from a single timepoint. For example, a protein may have lower measured levels than expected relative to its mRNA levels, either due to post-transcriptional regulation or difficulties in extraction or detection due to specific properties of the protein. It may be difficult to distinguish between these possibilities with single-sample comparison, but following mRNA and protein trends over many samples might be informative in defining the regulation for this gene, independent of any protein-specific measurement biases.

Moreover, regulatory mechanisms can be employed with precise timing that may be obscured in experiments in which only start- and end-points are measured. This is illustrated by an elegant study from Giraldez and colleagues (Bazzini et al. 2012), who measured the effects of the microRNA miR-430 on target translation and degradation. Prior to this study, it was unclear whether miRNA-mediated gene repression during vertebrate embryogenesis was primarily due to translational repression or mRNA degradation [reviewed in Djuranovic et al. (2011), Fabian et al. (2010)]. The authors performed RNA-seq and ribosome profiling at 2, 4, and 6 h post-fertilization (hpf) in zebrafish embryos. Strikingly, they found that miR-430 targets showed reduced translation at 4 hpf despite constant mRNA levels. By 6 hpf, both mRNA abundance and translation were reduced. The authors concluded that miR-430 first acts to prevent the translation of its targets before directing them for degradation. In this case, parallel, time-resolved, and genome-wide measurements were essential to determining causality that would have been masked by more distantly spaced timepoints. The authors were also aided by their choice of model system, which enabled study of miRNA-mediated repression in a natural context, rather than relying on mis-expression experiments, which might not always simulate physiological states.

## Observing natural dynamic processes unmasks diverse regulatory mechanisms

Developmental processes, including cell differentiation and embryogenesis, allow researchers to study gene regulatory mechanisms in the context in which they evolved. In contrast, commonly studied lab conditions tend to be unnaturally rich or harsh, which may be valuable in revealing strong regulation, but may not accurately represent any real physiological state. Meiosis and other developmental processes involve sequential unidirectional cell state changes, often driven by “waves” of gene regulatory changes (Chu et al. 1998; Jin and Neiman 2016). We leveraged this feature of meiotic differentiation to reveal distinct modules of temporal gene expression control.

Another valuable developmental program for the study of gene regulation is oogenesis, in which eggs mature in the absence of new transcription and thus rely heavily on post-transcriptional regulatory mechanisms [reviewed in Johnstone and Lasko (2001), Tadros and Lipshitz (2009), Vardy and Orr-Weaver (2007)]. Using RNA-seq and ribosome profiling, Orr-Weaver, Bartel, and colleagues (Kronja et al. 2014) identified broad changes in translational regulation during the transition from mature oocyte to activated egg in *Drosophila*. To further assess the mechanisms underlying this translational regulation, the same groups coupled poly(A) tail length profiling to parallel ribosome profiling and RNA-seq and found a strong correlation between poly(A) tail length and TE during oogenesis (Eichhorn et al. 2016). This correlation persisted into the first few embryonic cell divisions, but disappeared during gastrulation, suggesting that the use of this mechanism is confined to a precise time in development.

Another recent study described gene regulatory processes over a timecourse of induced neuronal development using RNA-seq, ribosome profiling, and polysome profiling (to measure differential translation of intact transcript isoforms) (Blair et al. 2017). The authors found some regulatory features that were employed only at certain stages of development. For example, long 3′ UTRs strongly repressed translation in differentiated neurons but had no effect in embryonic stem cells. Neuronally repressed transcripts were associated with the addition of putative structured elements and brain-specific miRNA binding sites in extended 3′ UTRs. While the developmental context and specific mechanisms differ, this finding is similar in concept to the LUTI-based mechanism in that transcripts with identical coding regions are differentially translated based on the inclusion or omission of cis-regulatory sequences (Chen et al. 2017; Cheng et al. 2018; Chia et al. 2017; Tresenrider and Ünal 2017).

These examples highlight an interesting feature of developmental gene regulation—different developmental processes may rely on different mechanisms to execute gene regulatory programs. Why is poly(A) length so important during oogenesis? Why do neurons encode extensive regulatory information in their 3′ UTRs when stem cells do not? Why are LUTI mRNAs abundant in meiotic differentiation? These questions emphasize the importance of studying diverse developmental processes if we are to uncover the full repertoire of gene regulatory strategies employed across biological systems.

## Conclusions

Here we argue for the value of gene regulatory studies that (1) measure expression globally, (2) compare multiple outputs measured in parallel, (3) span several timepoints, and (4) explore natural developmental processes. These four features are often interconnected—for example, it is possible to take many informative timepoints during developmental processes because they are inherently dynamic, comprised of a progressive series of cell state changes. We were guided in our study by a model from one well-defined instance of LUTI-based regulation. However, with the current computational power available, a pre-existing model may not be necessary for the future identification of common non-canonical regulatory mechanisms. Instead, such discoveries may simply involve an integrated and unbiased analysis of parallel features detected and measured in a single, well-constructed dataset.

Our understanding of molecular biology is built on hypothesis-driven research. Hypotheses provide a framework for experimental design and data interpretation. The growing use of genome-scale datasets for measurement of gene expression, however, has shifted the rules for designing informative experiments. Parallel global gene expression measurements produce massive datasets that can be analyzed productively without the need for a specific hypothesis. This is analogous to the type of experiments done following the development of the microscope, when simple observation of life led scientists to base future research directions on unusual features that they came across and wished to understand better. Then and now, technological advances allowed visualization of much more information than could be thoroughly analyzed by hypothesis-based approaches alone—an experiment could simply serve to generate questions that one did not previously know to ask. Our current age of genomic measurements offers the opportunity for us to develop new models based on what we observe, rather than requiring us to fit our data to existing models. While certainly not the first, our study, identifying widespread use of an unconventional mode of gene regulation during natural cellular development, provides a strong argument for seeking out apparent anomalies in large-scale datasets. These cases may not actually represent exceptions, and may instead reflect gaps in prior canonical models, which were necessarily built from analysis of a few examples rather than distilled from integrated genome-wide analyses.
